# Exudative pharyngitis possibly due to Corynebacterium pseudodiphtheriticum.

**DOI:** 10.3201/eid0302.970224

**Published:** 1997

**Authors:** P D Ellner


southern Arizona will be expanded in the coming years, and surveillance will continue for new dengue cases, imported or otherwise.

**David M. Engelthaler, T. Michael Fink, Craig E. Levy, and Mira J. Leslie**
Arizona Department of Health Services, Phoenix, Arizona, USA

### References

1. Sweeney K, Cantwell M, Dorothy J. The collection of *Aedes aegypti* and *Ae. albopictus* from Baltimore, Maryland. J Am Mosq Control Assoc 1988;4:381-82.
2. Donnelly J. *Aedes aegypti* in New Jersey. J Am Mosq Control Assoc 1993;9:238.
3. Bequaert J. *Aedes aegypti*, the yellow fever mosquito, in Arizona. Bulletin of the Brooklyn Entomological Society 1946;41:157.
4. Murphy D. Collection records of some Arizona mosquitoes. Entomological News 1953;14:233-8.
5. Richards CS, Nielsen LT, Rees DM. Mosquito records from the Great Basin and the drainage of the lower Colorado River. Mosquito News 1956;16:10-6.
6. Ferguson F, McNeel TE. The mosquitoes of New Mexico. Mosquito News 1954;14:30-1.
7. Smith HH, Janssen RJ, Mail GA, Wood SA. Arbovirus activity in southern Arizona. Am J Trop Med Hyg 1969;18:448-54.
8. McDonald JL, Sluss TP, Lang JD, Roan CC. Mosquitoes of Arizona. Tucson (AZ): University of Arizona Agricultural Experiment Station; 1973 Technical Bulletin No.: 205.
9. Service MW. Mosquito ecology: field sampling methods. New York (NY): John Wiley & Sons, 1976.
10. Rieter P, Amador M, Colon N. Enhancement of the CDC ovitrap with hay infusions for daily Monitoring of *Aedes aegypti* populations. J Am Mosq Control Assoc 1991;7:52-5.Treatment of Exudative Pharyngitis

**To the Editor**: The dispatch by Izurieta et al. (Emerg Infect Dis 1997;1:65-8) reporting exudative pharyngitis possibly due to *Corynebacterium pseudodiphtheriticum* was very interesting, especially with the resurgence of diphtheria in the former Soviet Union. However, I was somewhat surprised at the treatment received by the 4-year-old patient whose case is reported. Erythromycin is an effective antibiotic in diphtheria, but it is secondary in importance to diphtheria antitoxin.

The presence of a thick grayish white adherent pseudomembrane, adenopathy and cervical swelling, and low grade fever should certainly provoke a high index of suspicion of diphtheria, especially in a child who has not received pediatric immunization. The diagnosis of diphtheria is primarily made presumptively on clinical grounds and confirmed by the recovery of toxigenic *Corynebacterium diphtheriae* by the laboratory.

Antitoxin treatment cannot wait for laboratory confirmation. Prompt administration of antitoxin is important because diphtheria toxin binds rapidly and irreversibly to tissue sites. Delay in initiating antitoxin treatment is associated with increased incidence of myocarditis, paralysis, and death. Also, it would have been good practice to have placed this child in isolation until the diagnosis was established by the laboratory. The primary care physician in this case is indeed fortunate that the patient did not have diphtheria; the results could have been tragic.

**Paul D. Ellner, Ph.D.**
Professor Emeritus of Microbiology and Pathology, Columbia University College of Physicians and Surgeons

**Reply to P.D. Ellner:** We agree that diphtheria antitoxin should be administered promptly on the basis of a presumptive clinical diagnosis of respiratory diphtheria. Because laboratory confirma-tion may be delayed, the decision to treat with antitoxin and the dose of antitoxin must be based on the site and size of the diphtheritic membrane, the degree of toxicity, and the duration of illness (1,2).

Respiratory diphtheria is rare in the United States. From 1980 to 1995, only 41 cases were reported (zero to five cases in any given year) (3). With this low incidence, the likelihood that a patient with membranous pharyngitis has respiratory diphtheria is low. In addition, membranous pharyngitis could be associated with infections by other organisms such as streptococci, Epstein Barr virus, *Candida albicans*, *Borrelia vincenti*, *Herpes simplex* virus, *Arcanobacterium hemoliticum*, nontoxigenic *Corynebacterium diphtheriae*, and *Corynebacterium pseudodiphtheriticum* as in the case we reported (4-10).

The diagnosis and clinical management of exudative pharyngitis with a pseudomembrane in a country where diphtheria is extremely rare represent a dilemma for the practitioner. In weighing the benefits and risks of diphtheria antitoxin treatment, it is prudent to err on the side of using antitoxin.

**H.S. Izurieta,* P.M. Strebel,* T. Youngblood,† D.G. Hollis,* and T. Popovic***
Centers for Disease Control and Prevention, Atlanta, Georgia, USA; and †Rogers Pediatric Clinic, Rogers, Arkansas

### References

1. Farizo KM, Strebel PM, Chen RT, Kimbler A, Cleary TJ, Cochi SL. Fatal respiratory diphtheria due to *Corynebacterium diphtheriae*: case report and review of guidelines for management, investigation and control. Clin Infect Dis 1993;16:59-68.
2. American Academy of Pediatrics. Diphtheria. In: Peter G, editor. 1994 Red Book: Report of the Committee on Infectious Diseases. 23 ed. Elk Grove Village (IL): American Academy of Pediatrics, 1994:177-81.
3. Bisgard KM, Hardy IRB, Popovic T, Strebel PM, Wharton M, Hadler SC. Virtual elimination of respiratory diphtheria in the United States. Abstracts of the 36th Interscience Conference on Antimicrobials and Chemotherapy; 1996 Sep 15-18; New Orleans, LA; Abstract No. K166:280.
4. Wehrle PF. Diphtheria. In: Evans AS, Feldman HA, editors. Bacterial infections of humans. Epidemiology and control. New York: Plenum Medical Book Company, 1982:215.
5. Diphtheria. In: Krugman S, Katz SL, Gershon AA, Wilfert CM, editors. Infectious diseases of children. St. Louis (MO): Mosby Year Book, 1992:50.
6. MacGregor RR. *Corynebacterium diphtheriae*. In: Mandell GL, Bennett JE, Dolin R, editors. Principles and practice of infectious diseases, 4th ed. New York: Churchill Livingstone, 1995:870.
7. Kain KC, Noble MA, Barteluck RL, Tubbesing RH. *Arcanobacterium hemoliticum* infection: confused with scarlet fever and diphtheria. J Emerg Med 1991;9:33-5.
8. Robson JMB, Harrison M, Wing LW, Taylor R. Diphtheria: may be not! Communicable Disease Intelligence 1996;20:64-6.
9. Barksdale L, Garmise L, Horibata K. Virulence, toxinogeny, and lysogeny in *Corynebacterium diphtheriae*. Ann NY Acad Sci 1960;88:1093-108.
10. Izurieta HS, Youngblood T, Strebel PM, Hollis DG, Popovic T. Exudative pharyngitis possibly due to *Corynebacterium pseudodiphtheriticum*, a new challenge in the differential diagnosis of diphtheria: report of a case and review. Emerg Infect Dis 1997;3:65-8.

